# Youth telemental health and COVID-19

**DOI:** 10.1017/ipm.2021.27

**Published:** 2021-03-31

**Authors:** P. J. Archard, M. O’Reilly, S. Fitzpatrick, J. Fox

**Affiliations:** Child and Adolescent Mental Health Service (Young People’s Team), Leicestershire Partnership NHS Trust, Leicester, UK (Email: archardphilip@gmail.com); Department of Neuroscience, Psychology and Behaviour, University of Leicester, Leicester, UK; Leicestershire Partnership NHS Trust, Leicester, UK; Birmingham Women’s and Children’s NHS Foundation Trust, Birmingham, UK; Children’s Social Care and Early Help, Leicester City Council, Leicester, UK

Power *et al*.’s (2020) recent contribution highlights the anticipated increased demand for mental health support in young people and ways that the COVID-19 pandemic has impacted mental health across the age spectrum, disproportionately affecting younger populations. The need for greater investment in mental health is increasingly necessary as countries continue to cope with COVID-19 and its wide-ranging repercussions. We appreciate Power *et al*.’s contribution and support their argument that greater attention is needed for youth mental health, and significant research funds should be levied at improving knowledge of process, experience and outcomes. We would also emphasise the need for caution regarding the digital modalities they conclude their article foregrounding, and wanted to append some additional critical comments regarding youth telemental health during the pandemic.

Drawing on relevant literature, Power *et al*. highlight some of the advantages of these modes of delivery, drawing attention to their ‘youth friendliness’, ‘a democratisation of access’ to specialist and emerging therapies and widening access to services. Ostensibly, digital modes of delivery offer cost-effective ways to reach wider populations. And invariably it is vital to reflect on the role (and advantages) of these mediums given their present significance in enabling uninterrupted care, including where they may work just as well as attendance in person or be combined with face-to-face care, particularly if young people are understood as, as Power *at al*. refer to them, digital natives who may be inclined to look online for information and help.

Although the turn the pandemic necessitated away from face-to-face care to remote and digital delivery was in less-than-ideal circumstances, this is an overdue appraisal of professional and organisational reliance on clinic and office settings. As researchers and clinicians in the field of child and adolescent mental health, all serving various vulnerable groups (including young people who are looked-after, homeless and involved with youth justice services), we have, like others, had to adapt to develop our practice to this way of working. However, we would also adhere to a standpoint that the short-term acceptability of changes to working practices involving teleworking should be combined with the investigation of their longer term acceptability. Variable experiences of these mediums should not be overlooked, and time should be taken to consider who stands to benefit most and least from a reliance on their use.

It is also important to ask why there is resistance in the clinical world to these ways of working and to not reduce this to professional preference. Good clinical care *sometimes* needs to be face-to-face for very good reasons. Power *et al*. (2020) citing the comments of Wind *et al*. (2020) about professional resistance is important, but nonetheless glosses over this issue.

There is much that is different in establishing a treatment frame, and there can be significant challenges in working with young people suffering trauma and assessing patient affect, particularly dissociation, via video (Racine *et al*., 2020). As Power *et al*. acknowledge, the negative effects of the pandemic are unevenly distributed, which, for us, reinforces the need to question how teleintervention is navigated by clinicians in a context where social safety nets and community resources are compromised. Clinicians can adapt their practice on account of concerns regarding abuse and victimisation due to different circumstantial factors and ensure they routinely ask how things are going at home (Silliman Cohen & Bosk 2020). However, interpreting risk via video link or telephone can be anxiety provoking and can feel more like surveillance than support to young people and caregivers (Cowan *et al*., 2019), leaving clinicians feeling obliged to escalate any self-reported risk information and potentially increasing demand on crisis and urgent care services.

Alongside these clinical care factors, there are a plethora of issues to consider relating to connectivity and access to suitable technology and other factors outside the clinician’s and patient’s control, including not actually having a physical space to talk which is sufficiently private (Feijt *et al*., 2020, Zhai 2020). Multidisciplinary working within mental health services is as important as ever to ensure care remains comprehensive and consistent (O’Brien & McNicholas, 2020), yet remote working can mean social disconnectedness among staff. Boundaries between home and work are also less refined, with many clinicians managing increased household responsibilities and experiencing difficulties separating work from home life (Vogt *et al*., 2019).

At the centre of all this, though, is the young person, and it is necessary to be cautious when making assumptions that millions of young people who happen to be born from a certain date are likeminded in any meaningful way (Betton & Woollard 2019). It is not the case that young people acquire their digital literacy automatically or passively, and assumptions should not be made that a whole generation has access to digital technology and have the competencies to meaningfully engage with the content, even if it is designed to be youth friendly (O’Reilly *et al*., in press). Young people are not a homogenous group and can have mixed feelings about teleintervention, including desiring or needing time offline when engaging with mental health professionals and services. While many may have sufficient digital competency to engage with support online, it is not the case that all of them will want their care delivered that way. In a world where practice should be child- and youth-centred and young people should be empowered to make choices and have rights to make decisions, we argue that options should be available, and assumptions should not be made about their preferences.

Clearly, there is scope for services to increase their digital offer, and COVID-19 has helped to realise valuable lessons, but policy makers and commissioners should not be led to the impression that this comprises a pragmatic and economical solution. Instead, services should be supported to offer an integrated blend of care options, which account for clinicians’ experience, the preference of the young person, their family and caregivers, the nature of presenting problems and evidence, so that the right kind of care is delivered, through an appropriate modality.
